# Anesthetic efficacy of two administration regimens of obucaine hydrochloride gel for transnasal fiberoptic laryngoscopy: a single-center retrospective cohort study

**DOI:** 10.3389/fmed.2026.1818732

**Published:** 2026-08-04

**Authors:** Hua Chen, Shouyu Lin, Hua Zeng, Rong Zhang

**Affiliations:** Department of Outpatient Medicine, 900th Hospital of PLA Joint Logistic Support Force, Fuzhou, China

**Keywords:** administration regimen, obucaine hydrochloride gel, pharyngeal discomfort, retrospective cohort study, topical anesthesia, transnasal fiberoptic laryngoscopy

## Abstract

**Background:**

The primary objective of this study was to compare the comprehensive clinical tolerability and procedural performance, including intra-procedural pain, post-procedural pharyngeal discomfort, procedural efficiency, and safety, of nasal-only vs. combined nasopharyngeal anesthesia with obucaine hydrochloride gel for transnasal fiberoptic laryngoscopy (TFL). This study explicitly differentiated comprehensive clinical experience from pure analgesic efficacy, and made no *a priori* claim of superior anesthetic potency between the two regimens.

**Methods:**

This single-center retrospective study enrolled patients undergoing first-time TFL between June and December 2025, who were assigned to either nasal-only anesthesia (Group A) or combined nasopharyngeal anesthesia (Group B). 1:1 propensity score matching (PSM) was conducted to balance baseline confounding variables. The primary outcome was the incidence of moderate-to-severe post-procedural pharyngeal discomfort assessed within 2 h of TFL, a composite endpoint reflecting holistic patient experience and procedural feasibility. We explicitly acknowledged that this endpoint includes functional impairments inherent to pharyngeal topicalization and may structurally favor the nasal-only regimen. This endpoint was not designed to measure pure analgesic efficacy. Secondary outcomes included examination duration, operator-assessed procedural difficulty visual analog scale (VAS) score, patient-reported pain VAS score, and adverse events.

**Results:**

A total of 950 patients were initially enrolled (470 in Group A, 480 in Group B), and 420 patients per group were included in the final analysis following 1:1 PSM. Baseline characteristics were well-balanced between groups, with all *p* > 0.05 and standardized mean differences (SMD) < 0.1 for all covariates. Group A exhibited a significantly lower incidence of moderate-to-severe pharyngeal discomfort (28.33% vs. 52.14%, *p* < 0.001), shorter examination duration, and higher operator-assessed procedural smoothness VAS scores (reverse scoring: higher scores indicate lower procedural difficulty; all *p* < 0.001). After multivariable adjustment, no statistically significant difference in patient-reported pain VAS scores was observed between groups (*p* = 0.068), indicating comparable patient-reported pain intensity between the two regimens. Regarding safety, no systemic adverse events were observed in either group, and local adverse event rates were extremely low, with no statistically significant between-group difference detected in this cohort. No major safety signals were identified in this cohort.

**Conclusion:**

Among relatively healthy outpatients aged 18–60 years undergoing diagnostic-only TFL without anatomical abnormalities or concurrent therapeutic procedures, nasal-only administration of obucaine hydrochloride gel was associated with superior procedural tolerability and shorter objective examination time compared with the nasopharyngeal combined regimen. No significant between-group difference was observed in patient-reported pain intensity, and no major safety signals were detected in this cohort. No conclusion of safety equivalence can be drawn due to the limited sample size and follow-up duration. These findings provide real-world evidence to guide anesthesia regimen selection in this specific outpatient population, and generate hypotheses for future prospective, multicenter studies to confirm these results in broader clinical populations.

## 1 Introduction

Transnasal fiberoptic laryngoscopy (TFL) is a core diagnostic technology for otorhinolaryngological diseases, and is widely used for the etiological evaluation of symptoms including hoarseness, dry throat, and throat obstruction, as well as the diagnosis and follow-up of early laryngeal cancer, nasopharyngitis and other conditions (1).

Globally, more than 10 million TFL examinations are performed annually, and tertiary hospitals in China perform an average of 30–50 TFL procedures per day in otolaryngology outpatient clinics (2). However, the invasive operation of TFL examination can easily irritate the sensory nerve endings of the mucous membrane of the nasal cavity and throat, causing pain, nausea, gag reflexes and other discomforts. These adverse effects can reduce patient cooperation, lead to examination interruptions, compromise diagnostic accuracy, and increase medical resource utilization (3). Therefore, optimizing pre-operative topical anesthesia protocols is critical to enhancing the feasibility and patient experience of TFL.

Obucaine hydrochloride gel has emerged as the preferred topical anesthetic for TFL examination due to its rapid onset of action (2~3 min), high anesthesia potency (twice that of lidocaine), and low mucosal irritability (4, 5). However, considerable clinical controversy remained regarding its optimal administration range. Proponents of combined nasopharyngeal anesthesia argued that the pharynx was the core trigger zone for the pharyngeal reflex, and that additional anesthesia can suppress this reflex and reduce nausea (6). Opponents contended that pharyngeal anesthesia may excessively suppress physiological protective reflexes, leading to secretion retention and swallowing dysfunction, which in turn exacerbated pharyngeal obstruction and provided no significant analgesic benefit (2, 7).

Most existing relevant studies were small-sample, unblinded observational studies with low levels of evidence and single outcome measures, and they have neglected clinically relevant endpoints such as examination efficiency and procedural difficulty (8). Although prospective randomized controlled trials (RCTs) provide high-level evidence, their strict inclusion criteria may reduce the external validity of study findings. Retrospective cohort studies, which are based on real-world clinical practice data, can better reflect the real-world effectiveness of different anesthesia regimens in diverse populations and complement the limitations of prospective studies.

This single-center retrospective study aimed to compare the comprehensive clinical outcomes of nasal-only vs. combined nasopharyngeal anesthesia with obucaine hydrochloride gel in a selected cohort of outpatients aged 18–60 years undergoing diagnostic-only TFL. Patients with severe comorbidities, anatomical abnormalities, or concurrent therapeutic procedures were excluded. This observational study was designed to assess the association between anesthesia regimens and clinical outcomes, not to establish causal efficacy. We explicitly acknowledged the limitations of the retrospective design, including residual confounding.

## 2 Materials and methods

### 2.1 Study design

This study was a single-center retrospective cohort study, approved by the Ethics Committee of the 900th Hospital of Joint Logistics Support Force PLA (approval number: 2025-056). Given the retrospective analysis of de-identified medical record data, the ethics committee waived the requirement for informed consent from patients, and all data were anonymized to strictly protect patient privacy. All study procedures were conducted in accordance with the Declaration of Helsinki and its later amendments. The study was reported in accordance with the STROBE Statement. As an observational analysis of real-world clinical data, it can only assess associations between anesthesia regimens and study outcomes and cannot establish causal relationships.

### 2.2 Study objects and groups

#### 2.2.1 Source of cases

We retrospectively collected clinical data from patients who underwent first-time TFL in the otolaryngology outpatient clinic of our hospital between June and December 2025. Data were extracted from the hospital's electronic medical record (EMR) system, endoscopy database, anesthesia records, nursing assessment forms, and adverse event reporting system.

#### 2.2.2 Inclusion criteria

- First-time TFL recipients with complete examination records;
- Aged 18–60 years;
- Availability of complete clinical data including baseline characteristics, anesthesia regimen, outcome indicators, and adverse reaction records;
- Received obucaine hydrochloride gel as the sole anesthetic agent with a clearly documented administration regimen (either nasal-only or combined nasopharyngeal administration).

#### 2.2.3 Exclusion criteria

- Comorbid severe cardiac, pulmonary, hepatic, or renal insufficiency or other serious underlying diseases;
- History of tracheostomy, laryngopharyngeal foreign body, or malignant tumor causing difficult endoscope insertion;
- Acute pharyngitis, oral ulcers, or other conditions that could potentially interfere with pain assessment;
- Concurrent biopsy, foreign body removal, or other therapeutic procedures performed during endoscopy;
- Hypersensitivity to obucaine hydrochloride gel or related medications;
- Pregnant or breastfeeding women;
- Incomplete medical records (e.g., missing outcome measure documentation, unclear administration regimens).

#### 2.2.4 Grouping method

Patients were divided into two groups based on the anesthesia regimen they received in clinical practice. Group A (nasal-only topical anesthesia): obucaine hydrochloride gel was administered to the nasal cavity but not to the pharynx (only routine secretion clearance was performed), and the endoscope lens was coated with obucaine hydrochloride gel. Group B (combined nasopharyngeal topical anesthesia): obucaine hydrochloride gel was administered to both the nasal cavity and pharynx, and the endoscope lens was coated with the same gel. The dosage and lens coating method for both groups followed the standard clinical protocols documented in the medical records.

As this was a retrospective cohort study, the anesthesia regimen for each patient was selected by attending otolaryngologists based on their clinical experience and the patient's individual clinical and anatomical characteristics. Key factors influencing regimen selection included the patient's anticipated tolerance to pharyngeal anesthesia, gag reflex sensitivity, nasal anatomical narrowing, anxiety level, and the operator's clinical preference-factors that were strongly associated with both regimen allocation and study outcomes. However, due to the retrospective study design, these factors were not systematically documented in medical records and thus could not be included as matching variables in the PSM model. This may lead to residual confounding by indication, which is a major limitation of this study.

### 2.3 Main equipment and drugs

The endoscopic equipment used was an Olympus electronic nasopharyngoscope (model: ENF-VT2; diameter: 3.8 mm). The anesthetic agent was obucaine hydrochloride gel (specification: 10 ml/bottle containing 30 mg obucaine hydrochloride; Sinopharm approval number: H21023203; manufactured by Shenyang Oasis Pharmaceutical Co., Ltd.).

### 2.4 Data collection

#### 2.4.1 Baseline data

We collected data on age, gender, chief complaints (hoarseness, dry throat, throat discomfort, obstruction, etc.), degree of nasal mucosal inflammation (mild/moderate/severe, as documented in endoscopic reports), and presence of underlying diseases (hypertension, diabetes, etc.).

#### 2.4.2 Relevant data on anesthesia protocol

We recorded the route of administration, total dose of obucaine hydrochloride gel, and anesthesia preparation time (defined as the interval from the start of administration to endoscope insertion) for both groups.

#### 2.4.3 Outcome indicators

##### 2.4.3.1 Primary outcome: incidence of moderate-to-severe pharyngeal discomfort

Assessed based on nursing evaluation records completed within 2 h post-examination, using the following grading criteria:

Mild: pharyngeal numbness and swelling with preserved independent swallowing function;

Moderate: increased laryngopharyngeal secretions and subjective resistance during swallowing;

Severe: thick pharyngeal secretions, respiratory obstruction, loss of swallowing control, or forced interruption/termination of the examination.

The incidence was calculated as follows: (Number of moderate cases + Number of severe cases)/Total number of cases × 100%.

Note: this is a composite endpoint encompassing both subjective symptoms and functional impairments, some of which are inherent sequelae of pharyngeal topicalization. This endpoint may structurally favor the nasal-only group, and was designed to assess comprehensive clinical tolerability, not pure analgesic efficacy.

##### 2.4.3.2 Secondary outcome: examination duration

Defined as the “total duration from endoscope insertion into the nasal cavity to complete withdrawal” (recorded to the nearest second), as documented in the endoscopic report. This was the primary objective measure for assessing procedural efficiency.

##### 2.4.3.3 Secondary outcome: physician's operational difficulty VAS score

A 0~10 point scale was used, with a pre-defined reverse scoring rule. 0 points indicated extremely difficult procedure, 10 points indicated smooth operation without resistance, and higher scores corresponded to lower operation difficulty. Scores were recorded in real time by the attending operator immediately after the examination.

Note: this is a subjective auxiliary measure that has not been systematically validated for reliability and validity in the context of TFL procedural difficulty assessment. Due to the non-blinded retrospective design, this score may be subject to expectation bias and should only be used as a supplementary reference for procedural efficiency, not as core evidence of regimen superiority.

##### 2.4.3.4 Secondary outcome: patient-reported pain VAS score

A patient self-assessed score documented in the immediate post-examination nursing records, ranging from 0 to 10 points. A score of 0 denotes no pain, and 10 points denotes the worst imaginable pain; higher scores indicate greater pain intensity.

##### 2.4.3.5 Adverse reactions

We collected data on local adverse reactions (e.g., nasal irritation, dysgeusia) and systemic adverse reactions (e.g., dizziness, nausea, allergic reactions) occurring during the examination and within 24 h post-examination. Data were extracted from adverse event documentation and medical progress notes.

### 2.5 Statistical methods

Statistical analyses were performed using SPSS version 24.0 software. Continuous data are presented as mean ± standard deviation (x ± s), and between-group comparisons were made using the independent samples t-test. Categorical data are presented as numbers (percentages), and between-group comparisons were performed using the chi-square test; the rank-sum test was used for ordered categorical data. Fisher's exact test was used to compare adverse event rates given the extremely low event counts.

#### 2.5.1 Missing data handling

Missing data were handled using a pre-specified two-step process:

Exclusion of severe missing data: 45 cases with missing key outcome variables or anesthesia regimen documentation were excluded, as these data could not be reliably imputed.

Multiple imputation for minor missing data: for the remaining 978 cases, minor missing data in three baseline variables (age, nasal mucosal inflammation degree, underlying disease status, all missing rates < 5%) were handled using multiple imputation by chained equations with 20 imputations. Little's MCAR test confirmed the missing pattern was completely random (*P* > 0.05).

#### 2.5.2 Propensity score matching (PSM)

Propensity scores were estimated using a multivariate logistic regression model, with the dependent variable being the anesthesia regimen (1 = nasal-only anesthesia, 0 = nasopharyngeal combined anesthesia) and the independent variables including age, sex, complaint type, degree of nasal mucosal inflammation, and underlying disease status. A 1:1 nearest-neighbor matching strategy without replacement was employed, with a caliper width of 0.02 to minimize differences in propensity scores between matched pairs.

Comprehensive balance diagnostics were conducted after matching: (1) Standardized Mean Differences (SMD) were calculated for all matching variables, with SMD < 0.1 considered indicative of good balance; (2) Overlap assessment was performed to verify the distribution of propensity scores between the two groups; (3) Sensitivity analyses with different caliper values (0.01, 0.03) were conducted to evaluate the robustness of the PSM results.

#### 2.5.3 Multivariable regression analyses

Multivariable regression analyses was performed on the matched dataset to further adjust for minor residual imbalances in baseline covariates, a recommended approach to enhance the robustness of observational study findings.

After PSM, multivariable regression analyses were performed on the PSM-matched dataset to further adjust for minor residual baseline imbalances, with covariates identical to those included in. This approach was adopted to enhance the robustness of the results.

For the primary outcome (moderate-to-severe pharyngeal discomfort, binary variable), a multivariable logistic regression model was used. *E*-value analysis was conducted to quantify the potential impact of unmeasured confounding on the primary outcome.

For continuous secondary outcomes (examination time, patient pain VAS score, operator difficulty VAS score), multivariable linear regression models were used. Model assumptions (normality of residuals, homoscedasticity) were verified using the Shapiro–Wilk test and Q-Q plots. For VAS scores, ordered logistic regression was performed as a sensitivity analysis to confirm the consistency of results.

All multivariable regression analyses were performed as supportive sensitivity analyses to further adjust for minor residual baseline imbalances after PSM. These analyses did not establish causal relationships, and their results should be interpreted within the context of the overall observational study design. For continuous VAS outcomes, linear regression was used as the primary analytical method, consistent with standard clinical research practice. However, we acknowledged that VAS scores were bounded (0–10) and ordinal in nature, which may violate the strict assumptions of linear regression. Therefore, ordered logistic regression was performed as a sensitivity analysis to confirm the consistency of the findings. Full regression outputs were presented in Supplementary appendix 1.

## 3 Results

### 3.1 Screening and matching of study objects

From June to December 2025, a total of 1,023 patients underwent first-time TFL examination, forming the initial potential cohort. After excluding 73 ineligible cases (45 with severely incomplete records, 28 not meeting inclusion criteria), 950 patients remained as the eligible initial cohort. The full flowchart was presented in Supplementary appendix 2.

Multiple imputation was performed for the 3 baseline covariates with minor missing data (all missing rates < 5%) in the eligible initial cohort, generating an imputed dataset (*n* = 950). This imputed dataset was used for propensity score estimation and 1:1 PSM, resulting in 420 matched pairs (840 patients total), which constituted the final matched analysis dataset. The matching success rate was 89.36% for Group A and 82.68% for Group B. After matching, all baseline covariates had SMD < 0.1, indicating excellent baseline balance between the two groups, with no statistically significant differences (all *p* > 0.05, Table 1). No missing data remained in the final matched dataset after imputation and PSM. All primary and secondary outcome analyses were performed on the final matched analysis dataset. Sensitivity analyses using the complete-case dataset (excluding all cases with any missing data) yielded consistent results. (see Supplementary appendix 3).

**Table 1:** Baseline characteristics of the two groups before and after propensity score matching.

Baseline indicators	Before PSM			After PSM		
	Group A	Group B	SMD	Group A	Group B	SMD
	(*n* = 470)	(*n* = 480)		(*n* = 420)	(*n* = 420)	
Age (years), x ± s	40.11 ± 1.3	40.81 ± 0.9	0.063	40.31 ± 1.1	40.71 ± 0.8	0.037
Gender, *n* (%)			0.021			0.020
Male	281 (59.8%)	285 (59.4%)		252 (60.0%)	248 (59.0%)	
Female	189 (40.2%)	195 (40.6%)		168 (40.0%)	172 (41.0%)	
Main complaint, *n* (%)			0.085			0.078
Hoarseness	174 (37.0%)	181 (35.6%)		156 (37.1%)	148 (35.2%)	
Dry throat	116 (24.7%)	134 (26.4%)		105 (25.0%)	112 (26.7%)	
Throat discomfort	111 (23.6%)	125 (24.6%)		98 (23.3%)	103 (24.5%)	
Obstruction	69 (14.7%)	68 (13.4%)		61 (14.5%)	57 (13.6%)	
Degree of nasal mucosal inflammation, *n* (%)			0.052			0.041
Mild	319 (67.9%)	354 (69.7%)		286 (68.1%)	294 (70.0%)	
Moderate	114 (24.3%)	122 (24.0%)		112 (26.7%)	105 (25.0%)	
Severe	37 (7.8%)	32 (6.3%)		22 (5.2%)	21 (5.0%)	
Combined underlying diseases, *n* (%)	92 (19.6%)	97 (19.1%)	0.012	84 (20.0%)	79 (18.8%)	0.030

SMD, standardized mean difference. SMD < 0.1 indicates good balance between groups. All *P* > 0.05 after matching, indicating no statistically significant difference in baseline covariates between groups.

### 3.2 Comparison of major outcome indicators between the two patient groups

After 1:1 PSM, the incidence of moderate-to-severe pharyngeal discomfort in Group A (28.33%) was significantly lower than that in Group B (52.14%), a difference that was statistically significant (χ^2^ = 49.876, *P* < 0.001, Table 2). Multivariable logistic regression analysis revealed that after adjusting for age, gender, degree of nasal mucosal inflammation, and underlying disease status, nasal-only anesthesia remained a protective factor against moderate-to-severe pharyngeal discomfort (adjusted OR = 0.382, 95%CI: 0.295–0.495, *p* < 0.001). The *E*-value for the primary outcome was 3.21, indicating that an unmeasured confounder would need to be associated with both the anesthesia regimen and the outcome by a minimum of 3.21-fold to fully offset the observed association. While this magnitude of association was relatively uncommon in clinical observational studies, the possibility of residual confounding by unmeasured factors cannot be completely excluded, particularly given the retrospective design and the absence of systematic data on key clinical determinants of regimen allocation.

**Table 2:** Incidence of moderate-to-severe pharyngeal discomfort between the two groups after PSM [*n* (%)].

Group	Mild	Moderate	Severe	Incidence of moderate to severe discomfort
Group A (*n* = 420)	301 (71.67)	118 (28.10)	1 (0.23)	28.33%
Group B (*n* = 420)	201 (47.86)	216 (51.43)	3 (0.71)	52.14%
χ^2^	—	—	—	49.876
*P*-value	—	—	—	< 0.001

### 3.3 Comparison of secondary outcome indicators between the two groups

After matching, the examination time of Group A was significantly shorter than that of Group B, with a statistically significant difference (*p* < 0.001, Table 3). This objective measure indicated that the nasal-only obucaine hydrochloride gel anesthesia regimen was associated with better procedural tolerability and shorter objective examination time.

**Table 3:** Comparison of secondary outcome indicators between the two groups after PSM (x ± s).

Outcome indicators	Group A	Group B	*t*-value	*P*-value
	(*n* = 420)	(*n* = 420)		
Examination time (s)	65.81 ± 2.5	72.11 ± 4.5	−6.018	< 0.001
Operator difficulty VAS score (points)	8.71 ± .3	6.51 ± .7	22.893	< 0.001
Patient pain VAS score (points)	2.831 ± .25	3.051 ± .23	−2.642	0.008

The *P*-value for patient pain VAS score was 0.068 after adjustment for age, gender, nasal mucosal inflammation, and underlying diseases, with no statistically significant difference.

The operator difficulty VAS score (reverse scoring) was significantly higher in Group A than in Group B (*p* < 0.001, Table 3), consistent with the objective examination time result. However, given the subjectivity and unvalidated nature of this score, this finding should be interpreted with caution as an auxiliary reference only.

Univariate analysis revealed a statistically significant difference in patient pain VAS scores between Group A and Group B (*p* = 0.008, Table 3). However, after adjustment for confounding factors using multivariable linear regression, the difference was no longer statistically significant (adjusted β = −0.217, 95%CI: −0.449~0.015, *p* = 0.068). Ordered logistic regression sensitivity analysis yielded consistent results (*p* = 0.072), confirming no statistically significant difference in patient-reported pain intensity between the two regimens. These regression results were supportive of the primary PSM analysis and should be interpreted cautiously as observational associations rather than causal effects.

### 3.4 Comparison of adverse reactions between the two groups

No systemic adverse reactions were observed in either group during the study period or 24-h follow-up period. The local adverse reaction rates were extremely low in both groups, with no statistically significant difference between groups (Fisher's exact test, *p* = 0.144, Table 4). No major safety signals were observed in this study cohort. No cases of prolonged pharyngeal numbness lasting >1 h were observed in Group A, whereas two such cases occurred in Group B.

**Table 4:** Comparison of adverse reactions between the two groups after PSM [*n* (%)].

Adverse reaction type	Group A (*n* = 420)	Group B (*n* = 420)
Slight nasal irritation	2 (0.48)	3 (0.71)
Dysgeusia	1 (0.24)	4 (0.95)
Prolonged pharyngeal numbness >1 h	0 (0.00)	2 (0.48)
Total local adverse reactions	3 (0.71)	9 (2.14)
Systemic adverse reactions	0 (0.00)	0 (0.00)

Between-group comparison of total local adverse reaction rate: Fisher's exact test, *P* = 0.144.

## 4 Discussion

TFL is a cornerstone of otolaryngological diagnosis, but procedure-related discomfort remains a major barrier to patient acceptance and clinical efficiency. Optimizing topical anesthesia protocols is therefore critical to improving TFL practice (18). This retrospective cohort study compared nasal-only vs. nasopharyngeal combined anesthesia with obucaine hydrochloride gel in 840 propensity score-matched adult outpatients undergoing diagnostic-only TFL. The core findings were as follows: (1) Nasal-only anesthesia was associated with a significantly lower incidence of moderate-to-severe post-procedural pharyngeal discomfort; (2) Nasal-only anesthesia was associated with shorter objective examination time; and (3) No statistically significant difference in patient-reported pain intensity was observed between the two regimens after multivariable adjustment. No major safety signals were detected in either group.

The lower incidence of moderate-to-severe pharyngeal discomfort in the nasal-only group aligns with previous prospective studies (9, 10). However, it is critical to note that this primary endpoint was a composite measure incorporating functional impairments directly associated with pharyngeal topicalization, including swallowing difficulty and secretion retention. This introduced a structural bias favoring the nasal-only regimen, and the result should not be interpreted as indicating superior analgesic efficacy. We hypothesize that pharyngeal administration of obucaine hydrochloride gel, while temporarily inhibiting pharyngeal pain sensation, also impairs physiological protective reflexes of the throat, leading to increased laryngeal secretions and decreased swallowing coordination—effects that may themselves exacerbate post-procedural discomfort (10, 11). In contrast, nasal-only anesthesia targets only the nasal passage through which the endoscope passes, avoiding excessive interference with pharyngeal physiology while providing adequate anesthesia for the entry pathway.

The shorter objective examination time observed in the nasal-only group is the primary evidence of improved procedural performance. The higher operator-rated procedural smoothness VAS score (12, 13) was consistent with this finding, but this score was an unvalidated subjective auxiliary indicator and should be interpreted with extreme caution due to the non-blinded design and potential expectation bias. Two potential mechanisms may explain the shorter examination time: first, increased secretions and frequent swallowing in the pharyngeal anesthesia group may have caused blurred endoscopic vision and procedural interruptions; second, nasal-only anesthesia simplifies the anesthesia workflow by eliminating the pharyngeal administration step (14, 15). Notably, this study did not record the independent duration of pre-operative anesthesia preparation, so the time advantage of nasal-only anesthesia in this phase remains to be verified in prospective studies.

Regarding analgesic efficacy, the absence of a statistically significant difference in patient-reported pain intensity after multivariable adjustment suggested that the main source of pain during TFL was traction and irritation of the nasal mucosa, not the pharynx. This finding aligned with previous studies indicating that additional pharyngeal anesthesia does not further improve analgesia but may add unnecessary procedural steps (7, 16).

These results contributed to the ongoing debate regarding the optimal range of topical anesthesia for TFL. Proponents of combined nasopharyngeal anesthesia argued that pharyngeal anesthesia inhibited gag reflexes and reduced nausea (6), but our data suggested that this theoretical benefited may be offset by increased post-procedural functional impairments. Our findings were consistent with recent meta-analyses showing that limiting topical anesthesia to the nasal passage may improve overall patient tolerability without compromising pain control (9, 10, 17).

### 4.1 Strengths of the study

This study has several notable strengths. First, it is based on real-world clinical data from a large initial cohort of 950 patients, with a final matched sample of 840 patients, making it one of the largest studies comparing these two anesthesia regimens for TFL. The included population closely aligns with routine diagnostic TFL scenarios, conferring high external validity for this specific patient group. Second, we used 1:1 propensity score matching with rigorous balance diagnostics to minimize measured confounding, followed by multivariable regression analyses to further adjust for residual imbalances. Third, we included both objective procedural metrics (examination time) and patient-reported outcomes, enabling a holistic assessment of the two regimens.

### 4.2 Limitations of the study

First, residual confounding by indication remained the primary limitation. Although we adjusted for measured confounders using PSM and multivariable regression, several clinically relevant factors that influenced both anesthesia regimen selection and outcomes were not systematically recorded in electronic medical records, including patient anxiety level, gag reflex sensitivity, nasal anatomical narrowness, operator preference, and anesthesia administration technique. *E*-value analysis indicated that an unmeasured confounder would need to be associated with both the regimen and outcome by a minimum of 3.21-fold to fully offset the observed association. While this magnitude of association was relatively uncommon in clinical observational studies, the possibility of residual confounding cannot be completely excluded. All results presented in this study only reflected statistical associations, and no causal inferences can be drawn.

Second, the primary endpoint has inherent conceptual limitations. As a composite endpoint, it combines subjective symptoms and functional impairments that are inherent sequelae of pharyngeal topicalization, which structurally favors the nasal-only group. Additionally, the endpoint was not a validated patient-reported outcome measure (PROM), and its assessment based on nursing records introduces subjective bias, despite standardized training for nurses.

Third, the operator-rated procedural smoothness VAS score had significant limitations. This measure was not a validated tool for assessing procedural difficulty in TFL, and its subjectivity, compounded by the non-blinded study design, may have introduced expectation bias. This finding should only be interpreted as an auxiliary reference consistent with the objective examination time result, not as standalone evidence of regimen superiority.

Fourth, the safety assessment had important limitations. The follow-up window was only 24 h, so delayed adverse reactions could not be assessed. The overall adverse event rates were extremely low, which means the study was underpowered to detect meaningful between-group differences in rare adverse events. The study population was restricted to relatively healthy adults aged 18–60 years undergoing diagnostic-only TFL, so the safety findings cannot be generalized to broader populations, including elderly patients, those with severe comorbidities, anatomical abnormalities, or those undergoing concurrent therapeutic procedures. No conclusion of safety equivalence can be drawn from this study, and we can only confirm that no major safety signals were observed in this specific cohort.

Fifth, this is a single-center study, which may introduce center-specific bias. The findings may not be generalizable to other clinical settings with different patient populations or operator experience levels.

### 4.3 Clinical implications and future research

The findings of this study suggested that, among relatively healthy outpatients aged 18–60 years undergoing diagnostic-only TFL without anatomical abnormalities or concurrent interventions, the nasal-only obucaine hydrochloride gel anesthesia regimen may be a preferable option. This regimen was associated with better procedural tolerability and shorter objective examination time, with no major safety signals observed in this cohort. However, these findings were hypothesis-generating and cannot be extrapolated to broader clinical populations or other anesthetic agents. Well-designed prospective, multicenter randomized controlled trials are warranted to confirm these results in diverse populations-including elderly patients, those with comorbidities, and individuals undergoing therapeutic TFL and to validate the generalizability of these findings to lidocaine-based anesthesia regimens.

## 5 Conclusion

Among relatively healthy 18–60-year-old outpatients undergoing diagnostic-only TFL without anatomical abnormalities or concurrent therapeutic procedures, nasal-only obucaine hydrochloride gel anesthesia was associated with a significantly lower incidence of moderate-to-severe post-procedural pharyngeal discomfort, shorter examination time, and lower operator-rated procedural difficulty compared with nasopharyngeal combined anesthesia. Multivariable-adjusted analysis confirmed no significant between-group difference in patient-reported pain scores and intensity. No major safety concerns were identified in this specific cohort. The nasal-only anesthesia regimen was therefore associated with improved composite procedural tolerability in this population, delivering real-world evidence for clinical decision-making and hypothesis generation for subsequent prospective validation studies.

## Data Availability

The original contributions presented in the study are included in the article/Supplementary material, further inquiries can be directed to the corresponding authors.
