# Supplementary material for: Anesthetic efficacy of two administration regimens of obucaine hydrochloride gel for transnasal fiberoptic laryngoscopy: a single-center retrospective cohort study
Source: Front Med (Lausanne). 2026 Aug 4;13:1818732. doi: 10.3389/fmed.2026.1818732 (PMC13481512; doi:10.3389/fmed.2026.1818732)
Supplement: Supplementary file 1 [file Data_Sheet_1.docx]

**Appendix 1: Full Output of Multivariable Regression Analyses**

Appendix 1.1. Multivariable Logistic Regression for Primary Outcome (Moderate-to-Severe Pharyngeal Discomfort)

Dependent variable: Moderate-to-severe pharyngeal discomfort (binary: 1=yes, 0=no); Independent variables: Anesthesia regimen (Group A=1, Group B=0), age, gender (male=1, female=0), degree of nasal mucosal inflammation (moderate=1, severe=2, mild=0 as reference), underlying diseases (yes=1, no=0).

| **Variable** | **Coefficient**  **(β)** | **Standard Error**  **(SE)** | **Wald χ²** | **P-value** | **Odds Ratio**  **(OR)** | **95% Confidence Interval**  **(CI)** |
| --- | --- | --- | --- | --- | --- | --- |
| Anesthesia regimen (Group A vs Group B) | -0.963 | 0.142 | 45.729 | <0.001 | 0.382 | 0.295–0.495 |
| Age | 0.008 | 0.005 | 2.560 | 0.110 | 1.008 | 0.998–1.018 |
| Gender (male vs female) | 0.072 | 0.163 | 0.248 | 0.618 | 1.075 | 0.787–1.472 |
| Nasal mucosal inflammation (moderate vs mild) | 0.154 | 0.182 | 0.719 | 0.396 | 1.167 | 0.835–1.633 |
| Nasal mucosal inflammation (severe vs mild) | 0.216 | 0.321 | 0.457 | 0.499 | 1.241 | 0.683–2.254 |
| Underlying diseases (yes vs no) | 0.105 | 0.198 | 0.282 | 0.595 | 1.111 | 0.763–1.620 |
| Constant | 0.872 | 0.385 | 5.126 | 0.024 | 2.390 | 1.118–5.100 |

Model fit: Hosmer-Lemeshow test χ²=7.236, P=0.511, indicating good model fit. E-value for the primary outcome: 3.21.

Appendix 1.2. Multivariable Linear Regression for Continuous Secondary Outcomes

Appendix 1.2.1 Outcome: Examination Time (seconds)

Dependent variable: Examination time; Independent variables: Same as above.

| **Variable** | **Coefficient**  **(β)** | **Standard Error**  **(SE)** | **t-value** | **P-value** | **95% CI** |
| --- | --- | --- | --- | --- | --- |
| Anesthesia regimen (Group A vs Group B) | -6.325 | 0.987 | -6.408 | <0.001 | -8.258–-4.392 |
| Age | 0.052 | 0.036 | 1.444 | 0.149 | -0.019–0.123 |
| Gender (male vs female) | -0.428 | 1.135 | -0.377 | 0.706 | -2.652–1.796 |
| Nasal mucosal inflammation (moderate vs mild) | 1.256 | 1.268 | 0.991 | 0.322 | -1.234–3.746 |
| Nasal mucosal inflammation (severe vs mild) | 2.145 | 2.227 | 0.963 | 0.336 | -2.231–6.521 |
| Underlying diseases (yes vs no) | 0.876 | 1.372 | 0.639 | 0.523 | -1.815–3.567 |
| Constant | 75.218 | 2.678 | 28.087 | <0.001 | 69.962–80.474 |

Model fit: R²=0.087, adjusted R²=0.080; Shapiro-Wilk test for residuals W=0.987, P=0.123, indicating normality of residuals; Homoscedasticity test P=0.215, indicating homoscedasticity.

Appendix 1.2.2 Outcome: Patient-reported Pain VAS Score

Dependent variable: Patient-reported pain VAS score; Independent variables: Same as above.

| **Variable** | **Coefficient**  **(β)** | **Standard Error**  **(SE)** | **t-value** | **P-value** | **95% CI** |
| --- | --- | --- | --- | --- | --- |
| Anesthesia regimen (Group A vs Group B) | -0.217 | 0.112 | -1.937 | 0.068 | -0.449–0.015 |
| Age | -0.003 | 0.004 | -0.750 | 0.454 | -0.011–0.005 |
| Gender (male vs female) | -0.052 | 0.129 | -0.403 | 0.687 | -0.306–0.202 |
| Nasal mucosal inflammation (moderate vs mild) | 0.145 | 0.144 | 1.007 | 0.314 | -0.138–0.428 |
| Nasal mucosal inflammation (severe vs mild) | 0.218 | 0.253 | 0.861 | 0.390 | -0.279–0.715 |
| Underlying diseases (yes vs no) | 0.103 | 0.156 | 0.660 | 0.510 | -0.203–0.409 |
| Constant | 3.215 | 0.304 | 10.576 | <0.001 | 2.618–3.812 |

Model fit: R²=0.015, adjusted R²=0.008; Shapiro-Wilk test for residuals W=0.992, P=0.456, indicating normality of residuals; Homoscedasticity test P=0.327, indicating homoscedasticity. Ordered logistic regression sensitivity analysis: P=0.072, consistent with the linear regression result.

Appendix 1.2.3 Outcome: Physician’s Operational Difficulty VAS Score

Dependent variable: Physician’s operational difficulty VAS score (reverse scoring); Independent variables: Same as above.

| **Variable** | **Coefficient**  **(β)** | **Standard Error**  **(SE)** | **t-value** | **P-value** | **95% CI** |
| --- | --- | --- | --- | --- | --- |
| Anesthesia regimen (Group A vs Group B) | 2.187 | 0.108 | 20.250 | <0.001 | 1.975–2.399 |
| Age | 0.002 | 0.004 | 0.500 | 0.617 | -0.006–0.010 |
| Gender (male vs female) | 0.087 | 0.124 | 0.702 | 0.483 | -0.157–0.331 |
| Nasal mucosal inflammation (moderate vs mild) | -0.123 | 0.139 | -0.885 | 0.376 | -0.396–0.150 |
| Nasal mucosal inflammation (severe vs mild) | -0.187 | 0.244 | -0.766 | 0.444 | -0.666–0.292 |
| Underlying diseases (yes vs no) | -0.095 | 0.150 | -0.633 | 0.527 | -0.389–0.199 |
| Constant | 6.823 | 0.293 | 23.287 | <0.001 | 6.248–7.398 |

Model fit: R²=0.352, adjusted R²=0.347; Shapiro-Wilk test for residuals W=0.989, P=0.189, indicating normality of residuals; Homoscedasticity test P=0.278, indicating homoscedasticity.

**Appendix 2:** Total potential participants (n=1,023) → Excluded (n=73) → Eligible initial cohort (n=950) → Multiple imputation for minor missing data → Imputed dataset (n=950) → 1:1 PSM (caliper=0.02) → Final matched analysis dataset (n=840) → Final analysis.

**Appendix 3: Results of Sensitivity Analyses**

This appendix presents the results of sensitivity analyses performed to verify the robustness of the study findings. The sensitivity analyses included: 1) PSM with different caliper values; 2) Complete case analysis; 3) E-value analysis for the primary outcome.

**Appendix 3.1. PSM with Different Caliper Values**

To assess the impact of caliper value on PSM results, sensitivity analyses were performed using caliper values of 0.01 and 0.03 (original caliper=0.02). The main outcomes were compared between the two groups after matching with different caliper values.

| Caliper Value | Matched Pairs (n) | Group A (n) | Group B (n) | Moderate-to-Severe Pharyngeal Discomfort (Group A/Group B) | P-value | Examination Time (Group A/Group B, x±s, s) | P-value |
| --- | --- | --- | --- | --- | --- | --- | --- |
| 0.01 | 386 | 386 | 386 | 29.02% / 53.37% | <0.001 | 66.2±12.3 / 72.5±14.2 | <0.001 |
| 0.02 (original) | 420 | 420 | 420 | 28.33% / 52.14% | <0.001 | 65.8±12.5 / 72.1±14.5 | <0.001 |
| 0.03 | 442 | 442 | 442 | 27.83% / 51.58% | <0.001 | 65.5±12.7 / 71.8±14.7 | <0.001 |

Key Findings of PSM Sensitivity Analysis

Regardless of the caliper value (0.01, 0.02, or 0.03), the incidence of moderate-to-severe pharyngeal discomfort in Group A was significantly lower than that in Group B (all P<0.001), and the examination time of Group A was significantly shorter than that in Group B (all P<0.001).

After matching with different caliper values, the baseline covariates between the two groups remained well-balanced (all SMD <0.1), indicating that the choice of caliper value did not affect the balance of baseline characteristics.

The results of the sensitivity analysis confirm that the core findings of the study are robust and not affected by the choice of caliper value in PSM.

**Appendix 3.2. Complete Case Analysis**

A complete case analysis was performed to verify the impact of missing data on the study results. Complete case analysis included only patients with no missing data in all baseline variables, anesthesia regimen, and outcome indicators (n=933, Group A: n=452, Group B: n=481).

Key Results of Complete Case Analysis

Primary outcome: The incidence of moderate-to-severe pharyngeal discomfort in Group A was 27.88%, which was significantly lower than that in Group B (51.97%, χ²=45.321, P<0.001).

Secondary outcomes:
Examination time: Group A (66.1±12.4 s) vs Group B (72.3±14.6 s), t=-5.872, P<0.001;

Physician’s operational difficulty VAS score: Group A (8.6±1.4 points) vs Group B (6.6±1.6 points), t=21.987, P<0.001;

Patient-reported pain VAS score: After multivariable adjustment, P=0.071, with no statistically significant difference between the two groups.

The results of the complete case analysis were consistent with the main analysis (after multiple imputation and PSM), indicating that missing data did not significantly affect the study results and the findings are robust.

**Appendix 3.3. E-value Analysis for the Primary Outcome**

E-value analysis was performed to quantify the potential impact of unmeasured confounding on the primary outcome (moderate-to-severe pharyngeal discomfort). The E-value is the minimum strength of association that an unmeasured confounder would need to have with both the anesthesia regimen and the primary outcome to fully explain the observed association between the two.

Key Results of E-value Analysis

The E-value for the OR point estimate was 3.21, and the E-value for the lower bound of the 95% CI was 2.78, providing quantitative evidence that the primary outcome was relatively robust to unmeasured confounding of moderate clinical magnitude. In clinical practice, it is unlikely that an unmeasured confounder would have such a strong association with both the exposure (anesthesia regimen) and the outcome (moderate-to-severe pharyngeal discomfort). This indicates that the observed association between nasal-only anesthesia and lower incidence of moderate-to-severe pharyngeal discomfort is relatively robust to unmeasured confounding.

Overall Conclusion of Sensitivity Analyses

All sensitivity analyses (PSM with different caliper values, complete case analysis, and E-value analysis) yielded results consistent with the main study findings. This confirms that the core conclusions of the study—nasal-only anesthesia was associated with better procedural tolerability and improved procedural efficiency, with no statistically significant difference in patient-reported pain intensity—are robust and reliable.
